# Mesothelial-based cyst of the proximal jejunal mesentery: a rare cause of acute abdominal pain

**DOI:** 10.1093/jscr/rjag354

**Published:** 2026-05-09

**Authors:** Kiomara Oliveras Rivera, Valeria Matias Aponte, Carisa Jimenez Chaparro, Omar J Rovira Bellido

**Affiliations:** Medical Education, Mayagüez Medical Center, Mayagüez 00682, Puerto Rico; Universidad Autónoma de Guadalajara, Mayagüez Medical Center, Mayagüez 00682, Puerto Rico; Universidad Autónoma de Guadalajara, Mayagüez Medical Center, Mayagüez 00682, Puerto Rico; Department of Surgery, Mayagüez Medical Center, Mayagüez 00682, Puerto Rico

**Keywords:** mesothelial cyst, mesenteric cyst, jejunal mesentery, abdominal mass, abdominal pain, diabetes mellitus

## Abstract

Mesenteric cysts are rare benign intra-abdominal lesions, with mesothelial cysts representing an uncommon subtype. We present a 36-year-old female with acute abdominal pain and computed tomography scan with a large proximal jejunal mesenteric cyst. Surgical excision and en bloc resection were performed, and histopathology confirmed a benign mesothelial cyst. Postoperatively, the patient recovered uneventfully with improved glycaemic control, allowing discontinuation of antidiabetic therapy. This was considered an incidental finding.

## Introduction

Mesenteric cysts are an important differential diagnosis in the evaluation of abdominal masses. These intra-abdominal neoplasms are generally benign, with an estimated incidence of 1 in 100 000 to 250 000 [1]. The Perrot classification system facilitates their categorization according to their origin, which includes lymphatic, mesothelial, enteric, urogenital, mature cystic teratoma, and pseudocysts [2]. Mesothelial cysts can develop from the duodenum to the rectum, with the highest incidence observed in the ileum (60%), followed by the ascending colon (24%), and the retroperitoneum (14.5%) [3]. Approximately 40% of cases are identified incidentally, as symptoms are often nonspecific. Acute abdominal pain is the most prevalent manifestation in adults [4]. Diagnostic imaging is essential for diagnosis in the acute setting; nonetheless, definitive diagnosis relies on histopathological analysis.

This report presents the case of a female patient with acute abdominal pain, in whom a jejunal mesothelial cyst was diagnosed. Surgical intervention was offered, followed by histopathological confirmation of the diagnosis. Informed consent was obtained from the patient for the publication of this case report and associated images.

## Case report

A 36-year-old obese female with a past medical history of seizures and type 2 diabetes mellitus was transferred to our institution for evaluation of acute abdominal pain. The pain began suddenly without identifiable triggers, and she denied prior abdominal symptoms. She reported a three-year history of palpable cervical lymph nodes and B-symptoms (night sweats, low-grade fevers), and an unintentional 40-pound weight loss over one month. Given her symptoms and lymphadenopathy, a comprehensive lymphoma workup was completed and returned negative.

**Figure 1:**
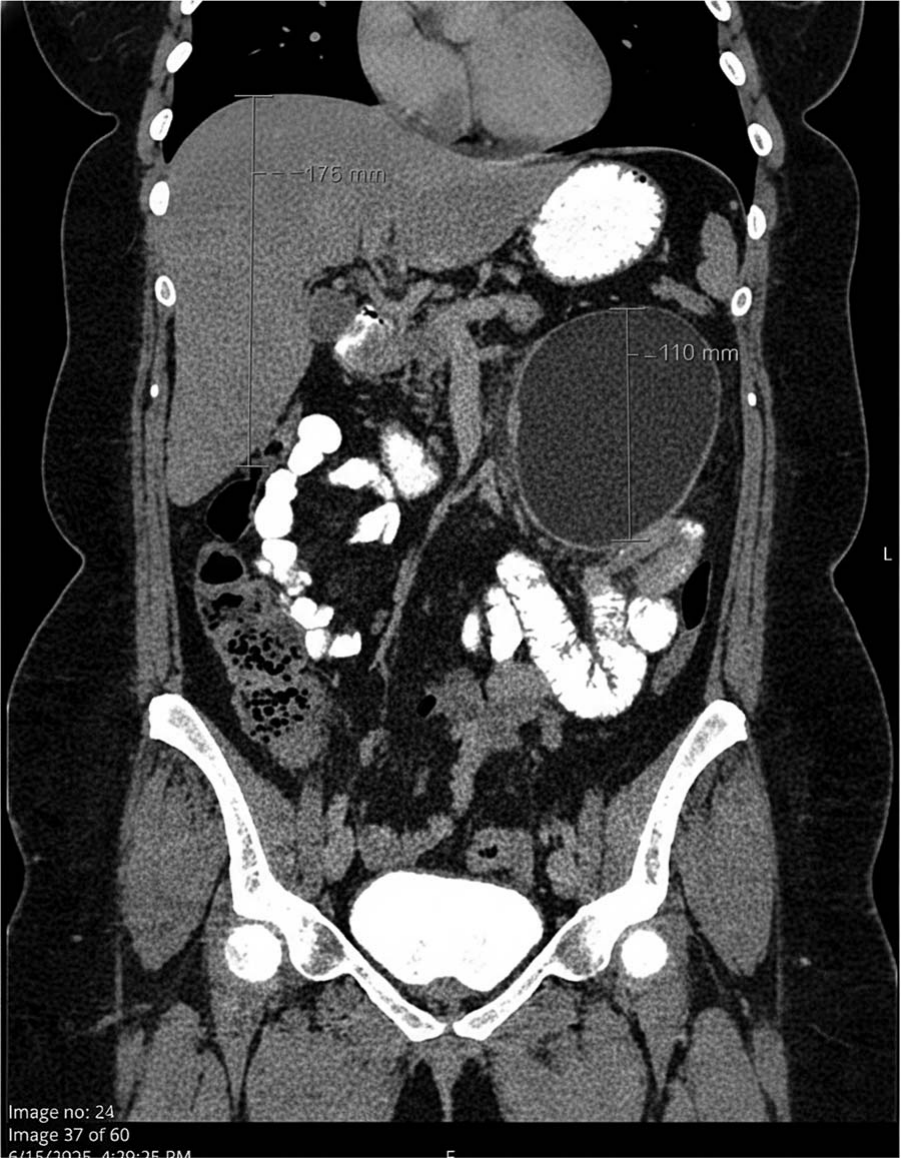
Coronal view showing a large, well-defined unilocular cystic lesion within the proximal jejunal mesentery, consistent with a mesenchymal cyst. Mild peripheral inflammatory stranding is noted, with no evidence of obstruction or ischemia.

**Figure 2:**
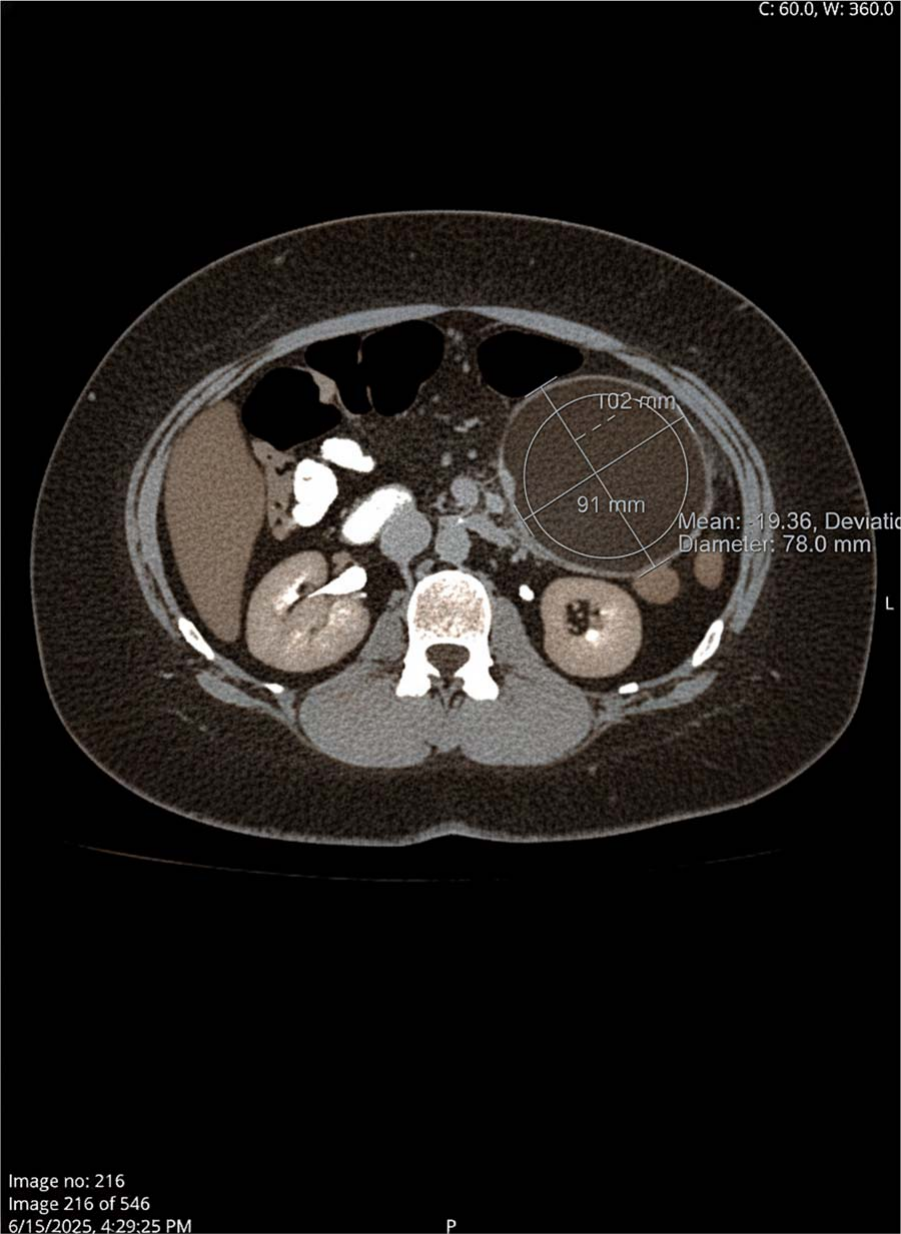
Axial view.

Physical examination revealed a palpable mass in the left upper quadrant. Laboratory workup revealed no leukocytosis and increased inflammatory markers (erythrocyte sedimentation rate 98 mm/h and C-reactive protein 17.10 mg/dL). Abdominopelvic computed tomography (CT) scan with IV contrast demonstrated a 10.2 × 10.0 cm unilocular, thick-walled cystic lesion within the proximal jejunal mesentery, with mild peripheral inflammatory fat stranding (Figs 1 and 2).

**Figure 3:**
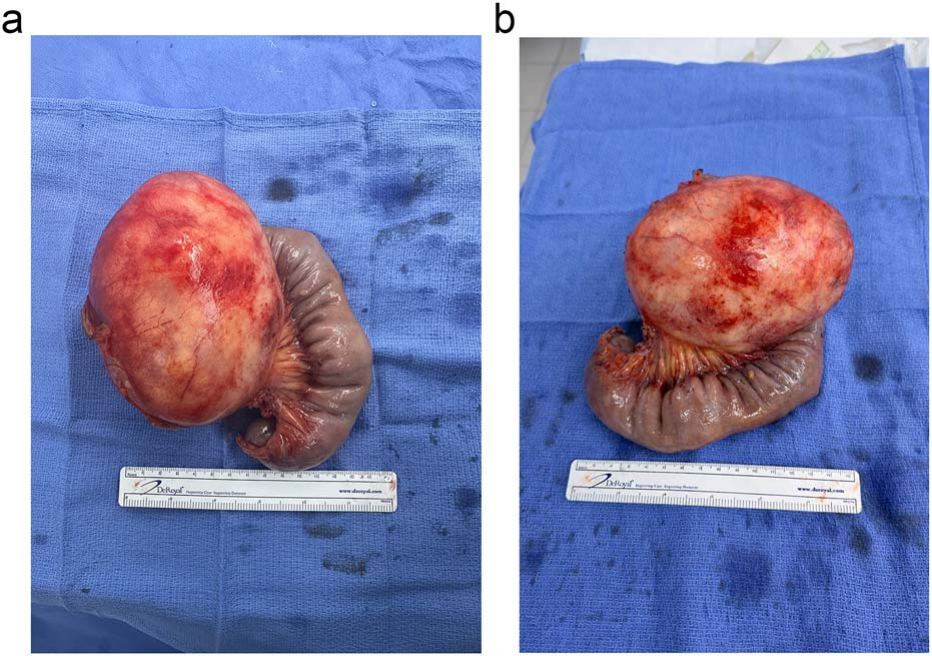
(a and b) Specimen of 11.0 × 11.0 cm proximal jejunal mesothelial based-cyst.

Patient was taken to the operating room for exploratory laparotomy which revealed a 13.5 × 10.0 × 10.0 cm proximal jejunal mesenteric mass 5 cm from the ligament of Treitz, tethering adjacent jejunum (Fig. 3a and b). En bloc resection of the cyst with adjacent jejunum was performed. Histopathology report confirmed a benign mesothelial-based cyst. Postoperatively, the patient recovered uneventfully and reported improved glycemic control without antihyperglycemic therapy.

## Discussion

Mesenteric cysts are rare, benign lesions, most commonly found in the small bowel (60% at the ileum), and the large bowel (24% in the ascending colon) [5]. These cysts are benign neoplasms that present asymptomatically or with non-specific symptoms, including abdominal pain, nausea, vomiting, and anorexia [5]. Asymptomatic mesenteric cysts are typically incidental findings on imaging studies [6]. CT scan is considered the gold standard for preoperative evaluation and cysts characterization. However, definitive diagnosis is established by histopathological examination [7].

The pathogenesis of mesenteric cysts remains unclear. They are thought to result from benign proliferation of ectopic lymphatic vessels that fail to connect with the central lymphatic system or from lymphatic channel malformations [7].

Additionally, lymphatic obstruction secondary to prior surgery, trauma, or neoplasm has been implicated [7]. Simple mesothelial cyst is one of the six types of histological features of mesenteric cysts. Cysts from mesothelial base origin are more frequently seen in young middle-aged females, which is consistent with our patient. It typically manifests with subtle nonspecific abdominal pain, which generally improves after surgical excision. Additional histological variants include cysts of lymphatic, enteric, and urogenital origin, as well as dermoid cysts and pseudocysts [8]. Mesenteric cyst size is highly variable. Although no formal classification system exists based solely on cyst dimensions, a review of the literature reveals that size ranges from millimeter to centimeters in diameter. The increase in size will be slow and progressive. The largest mesenteric cyst reported in literature measured 30.4 × 31.7 × 24.0 cm and weighed 16 kg in a 46-year-old woman. Large mesenteric cysts have an increased risk of potential blood flow impairment, which predisposes the patient to mesenteric ischemia. Moreover, large cysts may be a leading cause of obstruction if left untreated [5]. In our case report, despite the cyst’s size (13.5 × 10.0 × 10.0 cm), there was no evidence of obstruction, nor ischemia.

The treatment of choice for mesenteric cyst is R0 resection. Mesenteric cysts have a low recurrence rate after surgical excision, even though the exact percentage has not been described in the literature [8].

After cyst resection, at three-month postoperative follow-up, the patient demonstrated improved glycaemic control, with a glycated hemoglobin (HbA1c) of 5.2% compared with 6.9% preoperatively, despite discontinuation of antihyperglycaemic therapy. This observation is considered an incidental finding, and no causal relationship between cyst resection and glycaemic improvement can be established. Although no prior reports describe changes in glycaemic control following excision of a benign mesenteric cyst, it is possible that systemic inflammatory states may influence insulin sensitivity [9–11]. However, this remains speculative and warrants further investigation.

## Conclusion

Mesothelial-based mesenteric cysts are benign neoplasms; most found at the ileum of middle-aged females. To our knowledge, a mesothelial-based cyst with proximal jejunal mesenteric involvement has not been reported in the literature. Although abdominal ultrasound is a good diagnostic tool, we suggest a CT scan with IV and PO contrast for detailed cyst characterization and surgical planning. Treatment of choice for mesenteric cysts, regardless of the type, is R0 resection.
